# Genome sequence of *Lacticaseibacillus paracasei* ORD 0998 (CECT 30660), a probiotic bacterium for women’s health

**DOI:** 10.1128/mra.00849-24

**Published:** 2025-03-05

**Authors:** Andrea Rodenes-Gavidia, Vanessa Illescas, Juan Martínez-Blanch, Empar Chenoll, José Antonio Moreno-Muñoz, Jesús Jiménez López, Araceli Lamelas

**Affiliations:** 1 ADM BIOPOLIS, University of Valencia Science Park (Parc Científic de la Universitat de València), Valencia, Spain; 2 Laboratorios Ordesa, S.L., Parc Científic de Barcelona, Barcelona, Spain; Loyola University Chicago, Chicago, Illinois, USA

**Keywords:** genome, DNA sequencing, probiotics, CECT 30660, *Lacticaseibacillus paracasei*

## Abstract

*Lacticaseibacillus paracasei* is commonly isolated from dairy products and the microbiome of the human reproductive and gastrointestinal tract. We report the genome assembly of *Lacticaseibacillus paracasei* ORD 0998 (CECT 30660) with two contigs, having a size of 3,150,431 bp and a GC content of 46.33%.

## ANNOUNCEMENT

*Lacticaseibacillus paracasei,* a gram-positive bacterium, naturally lives in dairy products, such as fermented milk and cheese, and in the gastrointestinal tract and feces of humans and animals (1). These probiotic lactic acid bacteria are used as starter cultures and produce certain active proteins, such as P40 and P75, with anti-inflammatory and cell-growth-promoting activities (2, 3). *L. paracasei* ORD 0998 was originally isolated from a vaginal exudate sample collected with sterile swabs from a healthy woman and deposited in the Spanish Type Culture Collection (CECT) with reference CECT30660. CECT 30660 was isolated under sterile conditions by seeding exudate on MRS culture media, and it was grown on MRS (Merck KGaA, Darmstadt, Germany) medium and incubated anaerobically at 37°C for 17–24 h. This strain shows promise as a probiotic.

ORD 0998 genomic DNA was extracted using the MagAttract HMW DNA Kit (Qiagen, Germany). DNA quality/concentration was measured by Nanodrop (ThermoFisher Scientific, USA). Illumina and Oxford Nanopore Technologies (ONT) libraries were run using the same DNA preparation. For Illumina sequencing, Nextera XT DNA Library Prep Kit (Illumina, USA) paired-end library (2 × 300 bp) was constructed and sequenced on the Illumina MiSeq platform. ONT sequencing was performed following the fragmentation of genomic DNA with g-TUBE (Covaris, USA) to generate 12.5 kb fragments. Then, the library was built using the Native Barcoding Expansion Kit (EXP-NBD114) (ONT, UK) and sequenced on the ONT MinION platform with FLO-MIN106D flow cell R9.4.1.

Illumina sequencing generated 2,106,662 × 2 raw reads and 1,440,306 × 2 clean reads using Reformat (BBmap) (4) version 38.25 with a quality (Q) filter of 20 and a length (L) greater than 50 bp. ONT sequencing generated 1,246,194 raw reads with an *N*_50_ of 5,285 and 1,243,424 clean reads using NanoFilt (5) version 2.8.0 with a *Q* > 7 and *L* > 200 bp. Prior to quality control, Guppy (6) version 3.3.0 was used as base caller. Both data sets were assembled with Unicycler (7) version 0.4.8 and evaluated with Quast (8) version 5.0.0. Annotation was performed via NCBI PGAP (9) version 5.3.

16S rRNA was predicted by Barrnap (10) version 0.9 and annotated by BLASTn (11) version 2.12.0+ and the NCBI nt database. The best match was found to be *Lacticaseibacillus paracasei* CACC-566 (GenBank accession CP048003.1) with 100% identity and coverage, and an E-value of 0.0. Genomic identity was confirmed using the JSpeciesWS (12) server by a high ANIb score (98.26%) and highly aligned nucleotides (85.47%) with the type strain *Lacticaseibacillus paracasei* subsp. *paracasei* JCM-8130 (RefSeq ID: GCF_000829035.1). ANIb score higher than 95% indicates that the compared genomes belong to the same species. Default parameters were used for all software except where otherwise noted.

The complete genome of *L. paracasei* ORD 0998 has a coverage of 197.48× and contains two contigs, one circular chromosome (3,101,768 bp; 46.4% GC), and one circular plasmid (48,663 bp; 43.5% GC). The total genome length is 3,150,431 bp, *N*_50_ of 3,101,768 bp, and 46.3% of GC content, containing 2,878 protein-coding genes, 59 tRNAs, and 15 rRNAs.

## Data Availability

The Whole Genome Shotgun project of *L. paracasei* ORD 0998 (CECT 30660) has been deposited at NCBI under accession numbers CP174271-CP174272. The version described in this paper is the first version, CP174271-CP174272. The BioSample and BioProject accession numbers are SAMN43000418 and PRJNA1143368, respectively. The raw data are available in the Sequence Read Archive (SRA) under the accession numbers SRR30110408 and SRR30110409. The genome sequence assembly of *L. paracasei* ORD 0998 (CECT 30660) has been deposited in NCBI/RefSeq and NCBI/GenBank under accession number GCF_041283745.2.
